# Trauma in childhood and adolescence and impaired executive functions are associated with uncertain reflective functioning in mothers with substance use disorder

**DOI:** 10.1016/j.abrep.2019.100245

**Published:** 2019-12-30

**Authors:** Vidar Roald Kristiansen, Tore Bergby Handeland, Bjørn Lau, Kerstin Søderstrøm, Ulrika Håkansson, Merete Glenne Øie

**Affiliations:** a Department of Psychology, University of Oslo, Oslo, Norway; b Department of Research, Lovisenberg Hospital, Oslo, Norway; c Innland Norway University of Applied Sciences, Lillehammer, Norway; d Department of Research, Innlandet Hospital Trust, Lillehammer, Norway

**Keywords:** Parental reflective function, Cognition, Maternal, Aversive experiences, Hypomentalizing, Hypermentalizing

## Abstract

**Aims:**

Impairments in reflective functioning are known to have adverse effects on the ability to display sensitive parenting as a caregiver. Several factors are associated with impairments in reflective functioning, such as impaired executive functioning and experienced trauma. We investigated how these factors contribute to an impaired reflective functioning style, such as pathological certain or uncertain reflective functioning. Extreme scores on these two subscales reflect two kinds of impairments in reflective functioning. We assessed executive functions, reflective functioning, and trauma in 43 mothers diagnosed with substance use disorders (SUD).

**Methods:**

Certain or uncertain reflective functioning were assessed using the Reflective Functioning Questionnaire 8 (RFQ-8). Executive functions and trauma were assessed by administering various questionnaires, interviews and neuropsychological tests.

**Results:**

High uncertain reflective functioning was more than six times as common (odds ratio) in mothers reporting high amounts of trauma in childhood and adolescence compared with mothers reporting low amounts of trauma. Impaired executive functions were also significantly associated with high uncertain reflective functioning. Certain reflective functioning did, however, not show any significant associations.

**Conclusion:**

When the SUD mothers give information about relational trauma in childhood and adolescence, it might therefore be worth investigating and addressing the potential tendency to have an uncertain reflective functioning style.

## Highlights

• Uncertain reflective function in mothers with SUD are associated with trauma during childhood and adolescence.
• Impaired executive functions are significantly associated with uncertain reflective function.
• Certain reflective function is not associated with executive functions or trauma.

## 1 Introduction

### 1.1 Reflective function in mothers with substance use disorders

Some mothers with substance use disorders (SUD) manage to provide parenting and a good enough home environment to support their children's development. However, the majority of these mothers, in one way or another, are unable to, through a lack of necessary skills and/or social support (Siqveland, Haabrekke, Wentzel-Larsen, & Moe, 2014). Maternal SUD often impairs the quality of parenting and the mother's ability to relate to their child and increases the risk of maladaptive parenting practices like child abuse and neglect (Pajulo, Suchman, Kalland, & Mayes, 2006). As a group, mothers with SUD are often reported to have impairments in maternal reflective functioning (RF) (Pajulo et al., 2006, Suchman et al., 2005). RF is the manifestation of the capacity to mentalize (Suchman, Ordway, de las Heras, & McMahon, 2016). Parental reflective functioning (PRF) refers to the caregiver's capacity to reflect upon his/her own internal mental experiences as well as those of the child (Slade, 2005). When mothers struggle with PRF, this may have a negative effect on the growth of this ability in their children (Fonagy et al., 2004, Slade et al., 2005). Some studies (e.g. Levy & Truman, 2002) find that PRF mediate the association between several aspects of psychosocial development in children (e.g. social competence, withdrawal and attention) and maternal SUD. Further, mentalization-based parenting interventions are shown to improve behavior related to maternal caregiving (e.g. sensitivity to their child's needs, fostering of cognitive and social-emotional growth) and increased social functioning in older children (Suchman, DeCoste, Leigh, & Borelli, 2010).

Fonagy et al. (2016) developed the Reflective Functioning Questionnaire (RFQ), which measures *reflective style*. The RFQ is a relatively new instrument, which differs from more established clinical interviews measuring maternal RF, such as the Parent Development Interview (PDI) (Aber, Slade, Berger, Bresgi, & Kaplan, 1985). The PDI is a semi-structured interview with questions about the parents’ thoughts and feelings concerning their child and their affective experience of parenting. Interviews are recorded and transcribed verbatim for scoring. The PDI is producing a unidimensional scale for what many have argued could be a multidimensional concept (Gunderson & Choi-Kain, 2008). The RFQ assess RF through self-reporting. It is a brief, easy-to-administer multidimensional screening measure of RF (Fonagy et al., 2016). The RFQ measures the degree of certain RF (RFQc) and uncertain RF (RFQu) the respondents experience in relation to their own and other's mental states. The uncertain RF style, resembling hypomentalizing, prevents an adequate RF due to a concrete, rigid way of mentalizing, making the individual unable to consider complex ways of understanding others' and one's own mind. The certain RF style, resembling hypermentalizing, prevents adequate RF by making individuals too certain that their view of the world is the one and only truth, thereby implying no need to mentalize others' state of mind (Allen and Fonagy, 2006). High levels of either style of RF measured by the RFQ are considered as negative entities, as both reveal a failure to appreciate the opaqueness of mental states. In preliminary studies, RFQu is closely linked to psychopathology, while RFQc is not (Fonagy et al., 2016). Earlier analyses from our research group showed that a sample of mothers with SUD had significantly higher scores on the RFQu compared to the RFQc (Handeland, Kristiansen, Lau, Håkansson, & Øie, 2019). However, as far as we know no other study has assessed RF with the RFQ in mothers with SUD.

### 1.2 Association between executive functions and reflective functions

There are several factors all of which can contribute to the ability of a mother with SUD to mentalize. Executive functions are suggested to form the necessary, but not sufficient, basis for mentalization and RF (Stien and Kendall, 2004, Håkansson et al., 2018). The frontal lobe in the brain is important for executive functions (EF) (Stuss, 2011). EF refer to cognitive processes necessary for complex goal-directed behavior and adaptation to a range of environmental changes and demands that provide cognitive control over behavior, cognitions and emotions (Diamond, 2013). There is a consensus regarding viewing inhibition, working memory and cognitive flexibility as the core components of EF (Diamond, 2013). Individuals with SUD are shown to have disruptions in inhibition (Dolan, Bechara, & Nathan, 2008), working memory (Bechara, Martin, & Becker, 2004) and cognitive flexibility (Cunha, Nicastri, De Andrade, & Bolla, 2010). Recent studies points to the importance of tailoring treatment to better fit the cognitive functions of mothers with SUD (e.g. setting relevant goals, making procedures easier to understand) in order for therapy to be beneficial (Milligan, Usher, & Urbanoski, 2017). Recently, our research group found that SUD mothers with adequate PRF measured with the PDI exhibited significantly better EF compared to mothers with poor PRF, even after controlling for mental health status and intelligence (IQ) (Håkansson, Söderström et al., 2018a). However, as far as we know, no study has investigated the association between EF and different styles of RF (certain and uncertain) in mothers with SUD.

### 1.3 Associations between trauma, brain structure, executive functions and reflective functions

Studies have shown an association between physical and emotional maltreatment in childhood and adolescence, and later difficulties with RF (Abate et al., 2017, Van Schie et al., 2017). Being exposed to relational trauma over time may affect the brain and EF, which are important precursors for RF (Stien & Kendall, 2004). When activation of the stress response system persists over time (e.g. due to repeated exposure to psychosocial stress and trauma such as sexual, physical or emotional abuse, emotional neglect or bullying), both functional and structural changes may occur in the brain. The hypothalamic-pituitaryadrenal (HPA) axis may become overstimulated, as the brain fails to downregulate this activity through negative feedback mechanisms from prefrontal cortex (PFC) and hippocampus (Herman et al., 2016). This again may lead to an accumulation of cortisol, which has a neurotoxic effect on the brain (Tarullo & Gunnar, 2006). The chronically elevated level of cortisol may lead to especially adverse consequences on the development of EF in children and adolescents, as this may, over time, lead to the damaging of brain cells in a critical developmental phase (Frodl & O'Keane, 2013). One hypothesis is that stress-susceptible brain regions have unique periods of sensitivity to the effects of early stress (Andersen & Teicher, 2008). For example, childhood sexual abuse between 14 and 16 years have been associated with reduced frontal cortex gray matter volume which is important for EF (Andersen et al., 2008). Further, young adults who experienced childhood sexual abuse between either 3–5 or 11–13 years of age demonstrated maximal reductions in hippocampal volume (Andersen et al., 2008). Hippocampus is critical to declarative memory in humans (Van Petten, 2004). However, the research mentioned above are cross-sectional and do not provide evidence for cause-and-effect relationships.

In situations where the reflexive, automatic behavior is insufficient to achieve goals, individuals have to use and manipulate available information in the environment in order to be able to modify their behavior and achieve their goals. This operation puts high demands on both working memory and inhibition (Kluwe-Schiavon, Viola, Sanvicente-Vieira, Malloy-Diniz, & Grassi-Oliveira, 2017). A dysfunctional working memory and/or capacity of inhibition (i.e. EF difficulties) may therefore prevent an individual from taking in and manipulating information, hereby disabling RF as an affect regulation tool.

Results from our research group have shown that SUD mothers with low PRF measured with the PDI had significantly more experience of adversities in early childhood and latency, compared to mothers with high PRF (Håkansson, Watten et al., 2018). In longitudinal studies childhood trauma is correlated with earlier and more comprehensive substance abuse in the traumatized individual later on (Taplin, Saddichha, Li, & Krausz, 2014), which in turn may affects RF (Neger & Prinz, 2015). Experiencing relational trauma during childhood does sometimes result in individuals adapting an uncertain and unstable RF style (Allen, 2003, Fonagy and Bateman, 2008). Individuals may start to equate the outer reality with their inner mental reality (Fonagy & Target, 1996), to avoid mentalizing another person’s perspective. Individuals with this tendency are often intolerant of other, alternative perspectives (uncertain RF). Trauma might also lead to the certain RF, where the individual avoids considering the malicious intent of a verbally or physically violent figure, by basing their understanding, thoughts and feelings on fantasy and imagination, not by corresponding to reality (Fonagy et al., 2004). This might be explained by how these children experience traumatic relational events; children may react with a parasympathetically dominated “shut down” in order to mentally protect themselves from the event. Experiencing this neurological deactivation during relational trauma often leads to similar responses in later situations in which external triggers (e.g. witnessing intoxicated caregivers, witnessing verbally/physically aggressive caretakers) initiate the response (Schauer & Elbert, 2015). From this point of view, the earlier trauma is experienced in life, the more adverse the consequences may be on the development of RF.

While a large body of research supports the association between mothers with SUD and impaired PRF measured with the PDI, no research has assessed how different EF and experience of trauma in different stages of development may be associated with different styles of RF deficits (certain or uncertain RF) in mothers with SUD. Effective treatment or psychosocial interventions might differ for both types of RF impairments, and they might have different developmental and neurobiological underpinnings (Fonagy et al., 2016). Hence, the current study was tailored to provide more knowledge about the development and dynamics of RF in mothers with SUD.

#### 1.3.1 Aims and hypotheses

The first aim was to examine how, and to what degree, RF measured with the RFQ relate to trauma in different stages of development in mothers with SUD. We hypothesized that the amount of trauma experienced earlier in life would correlate more strongly with RF deficits (higher certain or uncertain RF) than the amount of trauma reported later on.

The second aim was to examine the association between different forms of EF and the two scales in the RFQ (certain or uncertain RF). Research shows that, on a general basis, EF and RF are correlated (Beer et al., 2006, Deater-Deckard et al., 2012). We therefore wanted to conduct exploratory analyses in order to investigate the potential correlation between EF and the two RFQ scales.

## 2 Methods

### 2.1 Participants

In this study, 43 mothers with SUD were recruited, either while being pregnant or during their postpartum period (Håkansson et al., 2018, Håkansson et al., 2018). Twenty-five mothers (58.1%) were recruited from different treatment facilities specializing in treating and taking care of pregnant women and families with infants, and a concurrent substance abuse disorder. Twelve of the women (27.9%) were recruited from outpatient clinics, and six mothers (14.0%) were recruited by health nurses working in the nearby municipalities. The recruitment period lasted for two years. The inclusion criteria were a former substance abuse problem and a current SUD. Eleven (25.6%) of the mothers received medically assisted rehabilitation and were prescribed either methadone or buprenorphine. The exclusion criteria were premature birth (<32 weeks and <1500 g), twins or triplets, multi-handicapped or a severely ill children, or an estimated full-scale IQ below 70 in the mothers. Children with neonatal abstinence syndrome (NAS) were not excluded. To the best of our knowledge, all the mothers were abstinent during the assessment period. For the majority of the mothers (62.8%), the child they participated in this study with was their first-born. Even though 16 of the mothers (37.2%) also had older children, only one (2.3%) had custody for the older child in this study. The rest of the children resided either in foster care facilities or with their father. When the assessment took place, the children’s age range in this study was from four to 18 months (M = 8.6, SD = 3.8). This is a suitable age to assess the mother–child relationship (Siqveland & Moe, 2014). Eleven (25.6%) of the children were born with NAS, and received medical treatment for this. During the inclusion period, 12 of the mothers (27.9%) lost custody of the child participating in the study. More demographic data are reported in Table 1.

**Table 1:** Sample characteristics.

*Demographic data*	Range	Mean(SD)	
Mother’s age	19–44	31.07 (6.37)	
Child’s age (months)	4–18	8.56 (3.79)	
Number of children	1–4	1.51 (0.80)	
Children in daily custody	0–2	1.00 (0.22)	

Civil status:	Number	Percentage	
Cohabitant	14	32.6	
Romantic partner	7	16.3	
Single	22	51.2	

*Highest completed education:*			
Did not complete Primary school	2	4.7	
Primary school	23	53.5	
High school	12	27.9	
Graduate or professional degree	6	4.7	

*Mental health data*(a)	Number	Percentage	
Current depression	16	37.2	
Previous depression	41	95.3	
Previous suicide attempt	29	67.4	
Self-harm	28	65.1	
Mani	16	37.2	
Bipolar	2	4.7	
Panic	26	60.5	
Agoraphobia	12	27.9	
Social phobia	21	48.8	
Obsession	11	25.6	
Compulsion	5	11.6	
OCD	1	2.3	
PTSD	29	67.4	
General anxiety	23	53.5	
Psychosis	18	41.9	
Drug induced psychosis	22	51.2	
Schizophrenia	0	0.0	
Anorexia	16	37.2	
Bulimia	8	18.6	
Binge eating	4	9.3	

*Substance abuse mother*(b)	Preferred (%):	Debut mean (SD)	Problematic % (N = 43)

Alcohol	16.3	13.09(2.98) (N = 42)	41.9
Abuse of prescribed medications	0	18.08(5.79) (N = 37)	74.4
Cannabis	14.0	16.21(4.39) (N = 42)	81.4
Amphetamines/Cocaine	37.2	17.82(4.42) (N = 38)	72.1
Opiates	32.6	20.28(5.95) (N = 25)	46.5
Poly-substance use	–	18.36(4.78) (N = 36)	74.4

Note. N = 43 SD = standard deviation.

(a) Mini-International Neuropsychiatric Interview 5.0.0 manual.

(b) European Addiction Severity Index (Europ-ASI) 5th edition.

### 2.2 Measures

#### 2.2.1 Mental health and use of psychoactive substances

The use of psychoactive substances was registered using the European Addiction Severity Index (Europ-ASI) 5th edition (McLellan et al., 1992), Norwegian version (Lauritzen, 2010). Europ-ASI is a semi-structured clinical interview, consisting of questions related to legal and illegal substance use, employment and support status, family, social relationships, as well as psychological and somatic issues. Validity and reliability has been reported to be satisfactory (Kessler et al., 2012, Kokkevi and Hartgers, 1995, McLellan et al., 1992). Screening for comorbid psychiatric disorders was done by administering the M.I.N.I. plus version 5.0.0, Norwegian version (Mordal et al., 2010, Sheehan et al., 2006). M.I.N.I is a diagnostic interview, consisting of 16 modules and covering 27 psychiatric diagnoses related to diagnostic criteria in ICD-10 (World Health Organization, 1993) and DSM-IV (American Psychiatric Association, 2000). M.I.N.I has a high inter-rater and test reliability (Rush, First, & Blacker, 2008).

#### 2.2.2 Reflective Functioning Questionnaire-8 (RFQ-8)

RFQ-8 is a self-report questionnaire designed for RF assessment (Fonagy et al., 2016). The RFQ measures the degree of certainty/uncertainty the respondents experience in relation to their knowledge about their own and others’ mental states. The eight items included in the RFQ-8 were all part of the original RFQ (54 questions) with findings providing preliminary evidence for its reliability and validity (Badoud et al., 2015, Fonagy et al., 2016). Examples of questions are: “*I always know what I feel*” or “*People’s thoughts are a mystery to me.*” Every question in the RFQ is to be answered on a Likert scale from 1, indicating that the respondent “strongly disagrees,” to 7, indicating that the respondent “strongly agrees.” All of the items that make up RFQ-8 are median-scored items. For instance, “I don't always know why I do what I do” is a median-scored item used in the calculation of both the certainty and uncertainty scale. To calculate the certainty score on this item, the scores were recoded to “3-2-1-0-0-0-0.” The highest score would be obtained by choosing alternative 1 – “strongly disagree,“ yielding a score of 3 on the certainty scale for this item. To calculate the uncertain RF score, the polarization would be the other way: 0-0-0-0-1-2-3. The highest score on the uncertainty scale would be obtained by choosing alternative 7 – “strongly agree,” yielding a score of 3. The RFQ-8 questionnaire is relatively new, and has no well-established or validated cut-off for clinically high scores on its scales (Luyten & Moulton-Perkins, personal communication, 2 June 2017). The total score for each of the scales in this study was calculated by adding together the scores and dividing by the number of items included. The cut-off was set at 1 for both scales. Scores above 1 were categorized as high, and scores below were categorized as low/normal. This cut-off was set based on the assumption that a mean score of at least one on either of these scales represents a marked mentalizing style.

#### 2.2.3 Executive functions (EF)

Neuropsychological assessments of maternal EF included an assessment of several EF. Raw scores were converted into t-scores. The following EF components were assessed:

Working memory: The Letter-Number Sequencing sub-test from the Wechsler Adult Intelligence Scale 4th edition (Wechsler, 2014) was used. Participants were given increasingly longer series of mixed numbers and letters at 1 s intervals. Participants had to repeat each series to the administrator using only the numbers first, from the lowest to the highest, followed by the letters in alphabetical order. Longer spans resulted in higher t-scores, reflecting higher capacity of auditory working memory.

Cognitive inhibition: To assess cognitive inhibition, the Color-Word Interference Test, Condition 3 from the D-KEFS (Delis, Kaplan, & Kramer, 2001) was used. In this test, participants had to repress the urge to read a colored word, and instead state the name of the color the word was written in as quickly as possible. Difficulties with inhibition were indicated by a longer time needed to complete the task as well as higher frequencies of errors, resulting in lower t-scores.

Cognitive flexibility: To assess cognitive flexibility, the inhibition-switching task in the Color-Word Interference Test, Condition 4 from the D-KEFS (Delis et al., 2001) was used. The task involved switching between reading the colored word and naming the color in which the word was written. Difficulties with cognitive flexibility were indicated by longer time used and number of errors committed during the task, resulting in lower t-scores.

#### 2.2.4 Traumatic Antecedents Questionnaire (TAQ)

The TAQ is a 41-item self-report questionnaire which assesses traumatic experiences during four different age periods: early childhood (0–6), school age (7–12), adolescence (13–18) and adulthood (Luxenberg, Spinazzola, & Van der Kolk, 2001). Traumatic experiences are gathered in 10 domains: (1) Competence, (2) Safety, (3) Neglect, (4) Separation, (5) Emotional Abuse, (6) Physical Abuse, (7) Sexual Abuse, (8) Witnessing, (9) Other Traumas, and (10) Alcohol and Drugs. The TAQ is scored by asking respondents to rate to what degree they experienced certain statements during each age period on a scale from 0 to 3, with 0 meaning “never or not at all,” 1 meaning “rarely” or “a little bit,“ 2 meaning “occasionally” or “moderately” and 3 meaning “often” or “very much.” To counteract the problem of multiple comparisons, we removed the least theoretically interesting variables and only did analyses on domains 3–10 on the TAQ, which assesses trauma/adverse events. We did not include the domains competence and safety, because these two domains assess adaptive functioning rather than trauma. For the purposes of the present study, a high/low categorical variable was created for each of the age periods. This was done by creating a “high group” (participants scoring between 2 and 3) and a “low group” (participants scoring between 0 and 2). This division is clinically meaningful and has been used in other studies (see Luxenberg et al., 2001).

### 2.3 Procedures

The participants were assessed, either in the treatment facility where they were living, or at home. The data was collected from a large battery of assessments, and only selected and relevant results are presented in this article. A clinical psychologist (UH), supervised by a specialist in clinical neuropsychology (MØ), collected all the data.

### 2.4 Statistical analyses

Before investigating the bivariate relationship between RFQ and EF, a Pearson correlation coefficient analysis was performed to investigate whether IQ was a confounding variable influencing the relationship between EF and RFQ. This analysis showed that IQ measures (nonverbal, verbal and full-scale IQ) were not significantly correlated to EF, RFQu or RFQc. Because IQ measures not was significantly associated with EF or RFQ, we did not control for IQ in the multivariate analyses. The magnitude of the correlations ranged between −0.23 and 0.14.

To examine the association between trauma and the RFQ scales, chi square tests were conducted, using Fisher’s exact probability test to calculate statistical significance. A Pearson correlation coefficient analysis was executed to investigate the correlation between the RFQ scales and EF. Two multiple regression analyses were used to investigate this correlation further: the RFQ scales were entered as dependent variables and the measurements of EF as independent variables. All executive functions variables were entered simultaneously in order to attempt to highlight the unique variance accounted for by each. A collinearity diagnostic was performed in order to investigate a potential problem of multiple collinearity (between executive functions measures). The specific collinearity diagnostic chosen was variance inflation factor (VIF).

## 3 Results

### 3.1 Aim 1 – RFQ scales and trauma

The RFQc scores varies between 2 and 0 and the mean score is 0.47 (SD = 0.49). The dichotomous RFQc variable therefore contains few participants categorized as belonging to the high group (n = 8), and many categorized as belonging to the low group (n = 35). The RFQu scores vary between 3 and 0 and the mean score is 1.28 (SD = 0.85). See Table 2 for descriptives on the dichotomous RFQu variables. The mean score for trauma (TAQ) in childhood varies between 0.22 and 2.33, with an average score of 1.45 (SD = 0.60). The mean score for trauma (TAQ) in school age varies between 0.67 and 2.56, with an average score of 1.69 (SD = 0.53). The mean score for trauma (TAQ) in adolescence varies between 0.56 and 2.61, with an average score of 1.91 (SD = 0.44). The mean score for trauma (TAQ) in adulthood varies between 0.72 and 2.67, with an average score of 2.00 (SD = 0.42). See Table 2 for descriptives on the dichotomous trauma variables.

**Table 2:** Fisher’s exact test showing the relationship between RFQu and trauma (TAQ).

Taq/trauma –scales(a)		RFQu = 0 (low)		RFQu = 1 (high)		Fisher (p-value)	Kendalls’ Tau-B	Gamma
		Frequency	%	Frequency	%			
Trauma in childhood	Low High	17 2	89.5 10.5	14 10	58.3 41.7	0.039	0.17*	0.34*
Trauma in school age	Low High	15 4	78.9 21.1	12 12	50.0 50.0	0.064	0.30	0.58
Trauma in adolescence	Low High	14 5	73.7 26.3	7 17	29.2 70.8	0.006	0.442**	0.744**
Trauma in adulthood	Low High	11 8	57.9 42.1	11 13	45.8 54.2	0.543	0.12	0.24

Note. N = 43, Fisher = Fisher’s exact probability test, 2-tailed.

*p < .05, **p < .01, 2-tailed, exact.

(a) Measured by the Traumatic antecedent questionnaire. Childhood (0–6), school age (7–12), adolescence (13–18) and adulthood.

As shown in Table 2, when analyzing the implications of when trauma was experienced, we found a significant association between the amount of trauma experienced in childhood and RFQu scores (Χ^2^(1) = 5.11, p = .039). The odds of high RFQu scores were 6.07 times higher for the mothers who had experienced high amounts of trauma in childhood compared to the ones who had not. There was also a significant association between the amount of trauma reported in adolescence and RFQu scores (X^2^(1) = 8.41, p = .006). The odds of high RFQu scores were 6.80 times higher for the mothers who had experienced high levels of trauma during adolescence compared to the ones who had not. A similar trend were found for trauma experienced in school age, but this association was not significant (X^2^(1) = 3.80p = .064). We did not find a significant association between RFQu scores and reported trauma in adulthood. There were no significant associations between the level of RFQc and the amount of trauma reported for the different age periods.

### 3.2 Aim 2 – RFQ scales and Executive Functions (EF)

See Table 3 for descriptives of EF. To investigate the association between RFQ and EF, a bivariate Pearson correlation coefficient analysis was performed, see Table 4 for a presentation of correlations. The following items correlated significantly with the RFQu: working memory (*r =* −0.47, p = .001) and cognitive flexibility (*r =* −0.40, p = .009). Hence, having an uncertain RF style (high scores on RFQu) was related to decreased executive functioning in our sample. None of the EF significantly correlated with RFQc.

**Table 3:** Descriptives of executive functions measures.

Executive functions	Range (t-scores)	Mean(SD)
Working Memory(a)	25–65	41.16 (8.82)
Inhibition(b)	20–65	39.81 (11.33)
Cognitive Flexibility(b)	20–63	35.16 (11.40)

*Note.* N = 43 SD = Standard deviation.

(a) Letter-Number Sequencing sub-test from the Wechsler Adult Intelligence Scale 4th Edition.

(b) Color-Word Interference Test, Conditions 3 and 4 from the Delis-Kaplan Executive Function System (D-KEFS).

**Table 4:** Pearson correlation coefficients between RFQ scales and executive functions.

Variables	1	2	3	4	5	6	7
1. Uncertainty(a)							
2. Certainty(a)	−0.60**						
3. Working memory(b)	−0.47**	0.19					
4. Cognitive flexibility(c)	−0.40**	0.27	0.64**				
5. Inhibition(c)	−0.19	0.28	0.72**	0.64**			

*Note.* N = 43.

*p < .05. **p < .01.

(a) Reflective Functioning Questionnaire, the 8-item version.

(b) Letter-Number Sequencing sub-test from the Wechsler Adult Intelligence Scale 4th Edition.

(c) Color-Word Interference Test, Conditions 3 and 4 from the Delis-Kaplan Executive Function System (D-KEFS).

As shown in Table 5, a multiple linear regression analysis, showed that all the EF explained 31.0% of the variance in RFQu (adjusted R^2^ = 0.26, p = .002). Working memory significantly predicted RFQu (β = −0.59, p = .006). Inhibition did not significantly predict RFQu, although there was a clear trend in that direction (β = 0.41, p = .051). Cognitive flexibility did not significantly predict RFQu. The total regression model for RFQc was not significant (adjusted R^2^ = 0.01, p = .393), nor were any of the partial regression coefficients (working memory, cognitive flexibility and inhibition). None of the specific variation inflation factors (VIF) indicated multicollinearity, even with a strict cut-off (VIF > 5).

**Table 5:** Multiple linear regression analysis with executive functions (independent) and RFQu (dependent).

	RFQu		
Variable	B	95% Cl (B)	β
Constant	3.14	[2.04, 4.23]	–
Working memory^(a)^	−0.06**	[−0.10, −0.02]	−0.59
Cognitive flexibility^(b)^	−0.02	[−0.05, 0.01]	−0.28
Inhibition^(b)^	0.03	[0.00, 0.06]	0.41
*R^2^*	0.31		
*F*	5.85**		

## 4 Discussion

### 4.1 The associations between trauma and reflective functions (RFQ)

We found no significant relationship between RFQc and the trauma variables. This lack of association will be discussed in the “limitations” section.

As expected, we found that trauma in early childhood and adolescent years were associated with reduced RFQu in the mothers with SUD. Trauma in school-age showed a similar trend, but it was not significant in our small sample. The odds ratio for high RFQu scores were more than 6 times higher for the mothers who had experienced high levels of trauma in early childhood and adolescence, compared to the ones who had not. The development of RF, hereunder the integration and proper regulation of emotions, occurs largely within the context of an adult’s attachment experiences throughout childhood (Ensink, Normandin, Plamondon, Berthelot, & Fonagy, 2016). Experiencing relational trauma within this period of time may impair the development of RF, creating the experience that the behavior of others is unpredictable and incomprehensible (Seligman, 2014). Research shows that the earlier trauma is experienced, the graver these consequences usually are (Kalmakis & Chandler, 2015). Experiencing trauma in childhood often leads to emotional dysregulation, especially in the absence of a secure/safe caregiver that can help the child comprehend and process the emotions. Further, this heightens the risk of the later development of psychiatric disorders characterized by emotional dysregulation as a core feature (anxiety and mood disorders, see Dvir, Ford, Hill, & Frazier, 2014). It is also shown that these individuals – with little experience in recognizing and relating to emotions – more frequently resort to the use of substances as a way of regulating emotions during adolescence (Garland, Pettus-Davis, & Howard, 2013).

During early adolescence, executive abilities and mentalizing abilities increase (Valle, Massaro, Castelli, & Marchetti, 2015). Puberty represents a period of synaptic reorganization and as a consequence it is possible that the brain is more sensitive to input in the realm of EF and social cognition (Blakemore & Choudhury, 2006; see also Andersen et al., 2008). Through maturational processes in adolescence, brain processing is seen to become more efficient and effective (Steinberg, 2008). Adverse life experiences in adolescence may negatively affect the development of EF (McEwen and Gianaros, 2010, Sheridan et al., 2012). Adolescents who experience traumatic stress and develop post-traumatic symptoms secrete higher levels of glucocorticoid cortisol that may have a neurotoxic effect on the brain (Carrion & Wong, 2012). As a consequence of weakened EF, as adults they may strongly disagree with items on the RFQ because they have difficulties in reflecting on their own inner mental states, reflecting uncertain RF (RFQu). Further, additional harmful factors (e.g. substance abuse and a substance abusing social network) may potentially accompany trauma during this stage of life. When exposed to repeated trauma, resorting to “self-medication” in order to cope with psychological distress is a known phenomenon (Garland et al., 2013). Our sample of mothers with SUD debuted relatively early with both the use and abuse of substances. The onset of substance abuse as a result of trauma during adolescence may disturb the developing brain during this critical period (Selemon, 2013).

In line with our hypothesis, we found no significant association between trauma experienced in adulthood and RFQu. One explanation may be that trauma experienced in adulthood is not as harmful for the development of RF as trauma experienced earlier in life, because in adulthood this ability has largely been developed.

### 4.2 The associations between EF and Reflective Functions (RFQ)

We found no significant relationship between EF and RFQc. This lack of association will be discussed in the “limitations” section. Working memory was the only variable that was significantly associated with RFQu in both the multivariate regression analysis and the Pearson correlation analysis. It was also the most significant variable in both analyses, indicating that low working memory capacity was associated with higher (impaired) scores on RFQu. Results concerning working memory are in line with contemporary research in the field of RF. Our sample turned out to have a lower than normative average score on working memory. Difficulties in working memory may lead to difficulties in focusing attention over time and focusing attention on only goal-relevant stimuli when facing situations evoking high levels of affect (Hofmann, Schmeichel, & Baddeley, 2012). Having a well-functioning working memory enables individuals to effectively contain complex mental states of self and others, and can therefore be viewed as a necessity in order to use RF.

Deficits in cognitive flexibility was significantly correlated with higher (impaired) RFQu scores in the Pearson correlation analysis. The significant association between cognitive flexibility and RFQu in our sample can be explained by how cognitive flexibility enables the mothers to shift focus back and forth between themselves and their children. Mental flexibility is also important for the ability to regard oneself from the outside and the child from within, something which is important for the ability to mentalize and for proper RF.

Finally, higher levels of inhibition showed a clear trend towards being significantly associated with higher levels of RFQu in the multivariate regression analysis. Inhibition is shown to play an important part in self-regulation through the active inhibition of behaviors such as impulse and input that are not in accordance with one’s goals (e.g. Berkman et al., 2011, Von Hippel and Gonsalkorale, 2005). A possible “goal” of an individual with high level of RFQu may be to avoid mentalizing other’s perspective, since they are often intolerant of other, alternative perspectives. A plausible explanation for our finding may thus be that higher levels of inhibition actually make these individuals more able to block other perspectives from entering their minds, and in doing so maintain or even strengthen their (impaired) uncertain RF.

## 5 Limitations

### 5.1 General limitations

Neither TAQ nor RFQ have been used extensively in research (Spinazzola, Blaustein, Kisiel, & van der Kolk, 2001), and no well-validated clinical cut-offs have been set (Luyten & Moulton-Perkins, personal communication, June 2nd, 2017). The cut-offs in our sample are therefore to be considered as preliminary. Further, our study was cross-sectional, and thus we cannot infer causality based on our findings. In order to investigate the causal relationships we are suggesting, a longitudinal study of these variables is needed. The children’s age-range was from four to 18 months and it is possible that this variation in the child's age may have affected the RF results somewhat. Finally, the mothers had several mental health diagnoses that may also have affected the results. This, however, is common in people with SUD and makes the results more representative of the group. Both poor EF (dependent variable) and poor RF (independent variable) increase the chances of developing poor mental health and psychopathology (Katznelson, 2014, Snyder, 2013). Therefore, because both EF and RF may be causal factors in the development of poor mental health, including mental health as a control variable in the regression analyses, may have produced overcorrected, anomalous and counterintuitive findings concerning the relationship between EF and RF.

### 5.2 The limitations of RFQc in our analyses

No significant associations were found between RFQc and trauma experienced in any of the age spans. The low number of mothers having high scores on RFQc (n = 8) caused several cells in the chi square tests to have expected counts of less than 5. This was partly corrected for by the fisher’s exact test, but a larger sample is needed to test traumas association with RFQc. However, our non-significant association between RFQc and trauma is in accordance with earlier preliminary studies finding RFQu closely linked to psychopathology, while RFQc was not (Fonagy et al., 2016). Low to moderate levels of certain RF seem to be somewhat protective against psychopathology (relating positively to anger control and negatively to trait anger), at least when found in non-clinical samples (Fonagy et al., 2016).

Also, no significant associations were found between RFQc and EF. Both the restricted variability (compared with RFQu) and the positively skewed distribution (with nearly 35% scoring 0) of the RFQc created artificially low correlations and standardized regression coefficients in analyses with the EF measures. Consequently this also made the weak trends less significant, increasing the chances of making a type II error.

The lack of association between RFQc and both trauma and EF may therefore reflect limitations in our data, rather than a real lack of association between them. It would hence be of great interest to investigate the relationship between RFQc and trauma and EF in a sample with both higher levels and a wider range of RFQc to properly assess this relationship.

## 6 Clinical implications

Our results suggest that experiencing trauma in early childhood and adolescence is an especially vulnerable age span for the development of uncertain RF in this group of mothers with SUD. When the SUD mothers give information about relational trauma in childhood and adolescence, it might therefore be worth investigating and addressing the potential tendency to hypomentalize. Research shows that substance abuse and experiencing trauma in adolescence often leads to further psychological distress (Garland et al., 2013) and social isolation (Chou et al., 2011, Cook et al., 2005). For example, adolescents exposed to repeated trauma have been found to have lower school attendance (DeSocio & Hootman, 2004). Corrective experiences and feedback are necessary in facilitating the development of RF after a teenage period characterized by trauma (Bateman and Fonagy, 2010, Choi-Kain and Gunderson, 2008). Individuals spending vast amounts of time by themselves during the adolescent period may not be exposed to a sufficient amount of social interaction to acquire well-functioning RF. A theoretically efficient way of creating a social setting that facilitates this type of corrective experience is group therapy. This could potentially increase both the quality and amount of social interaction with peers. Based on our results, it might be necessary to improve EF (working memory, inhibition and cognitive flexibility) in order to effectively implement a mentalization-based therapy successfully if the dominant style of mentalizing is an uncertain one. However, the results of the long-term effectiveness of interventions targeting increased EF are ambiguous, and not always found in follow-up studies (see Melby-Lervåg & Hulme, 2013).

## CRediT authorship contribution statement

**Vidar Roald Kristiansen:** Conceptualization, Formal analysis, Writing - original draft, Writing - review & editing. **Tore Bergby Handeland:** Conceptualization, Formal analysis, Writing - original draft, Writing - review & editing. **Bjørn Lau:** Writing - review & editing, Supervision. **Kerstin Søderstrøm:** Conceptualization, Funding acquisition, Writing - review & editing. **Ulrika Håkansson:** Conceptualization, Data curation, Project administration, Writing - review & editing. **Merete Glenne Øie:** Conceptualization, Project administration, Writing - review & editing, Supervision.

## Declaration of Competing Interest

The authors declared that there is no conflict of interest.
